# Supplementary material for: Rapid nongenomic estrogen signaling controls alcohol drinking behavior in mice
Source: Nat Commun. 2024 Dec 30;15:10725. doi: 10.1038/s41467-024-54737-6 (PMC11686278; doi:10.1038/s41467-024-54737-6)
Supplement: Supplementary file 1 — Supplementary Information [file 41467_2024_54737_MOESM1_ESM.pdf]

Supplementary Figures

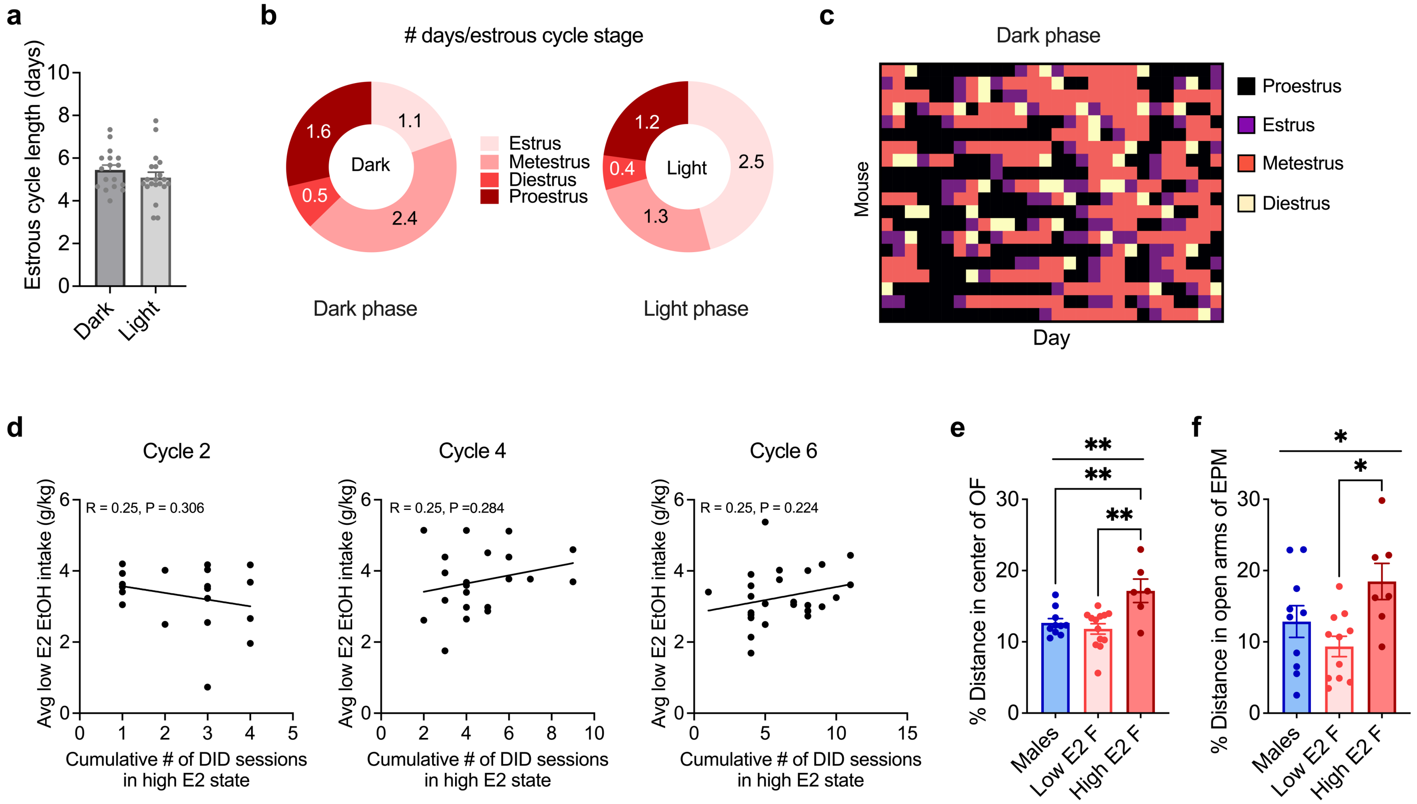

Supplementary Figure 1: Estrous cycle determined by vaginal cytology during the light and dark phases of the light-dark cycle (related to Fig. 1). a–c) Estrous cycle monitoring in naïve female mice in Fig. 1b-c. a) Average estrous cycle length of individual naïve female mice as measured by vaginal cytology daily 2 hour into was not different in dark vs. 2 hours into the light phase of the light-dark cycle (Dark cycle mice Ns = 17, light cycle mice N's = 20). b) Pie charts showing the average distribution of estrous cycle stages of mice in a. c) Heat map showing estrous cyclicity of individual mice across three weeks of tracking during the dark phase of the light-dark cycle, with each row representing one mouse and each color-coded cell on the x-axis representing estrous cycle stage on that day. Estrous cycle becomes stable across the first several weeks and then remains stable for months. d) The number of cumulative EtOH drinking days in a high ovarian E2 state did not affect subsequent drinking levels in a low ovarian E2 state at any point (Cycle 2: N's = 19; Cycle 4: N'2 = 21; Cycle 6: N's = 26). e-f) % Distance traveled in the center of the open field (OF; e, N's = 10 male, 13 low E2 females, 7 high E2 females) and in the open arms of the elevated plus maze (EPM; f; N's = 10 male, 11 low E2 females, 7 high E2 females) are consistent with % time spent in these compartments shown in Fig. 1i,l. *P < 0.05, **P < 0.01; one way ANOVA main effects of group, post hoc t-tests with H-S corrections as indicated. Data are presented as mean values +/- SEM. Detailed statistics are provided in Supplemental Table 1. Source data are provided as a Source Data file.

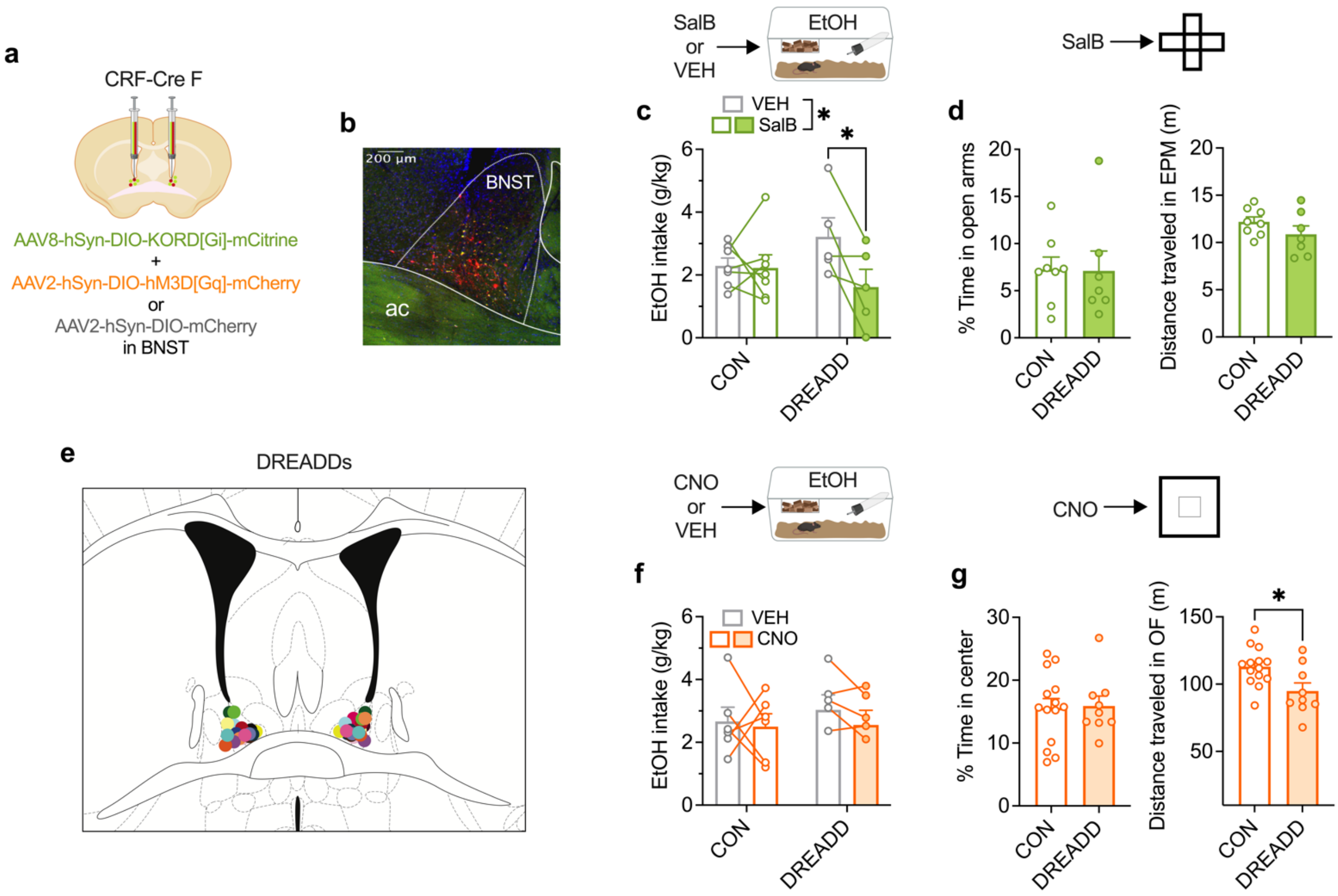

Supplementary Figure 2: BNST^{CRF} neurons are necessary for binge alcohol consumption. a-g) Chemogenetic manipulation of BNST^{CRF} neurons during EtOH Drinking in the Dark (DID) and avoidance behaviors. a-b) Schematic (a; Biorender license: KW27JLIRYP) and representative image (b) for viral strategy to bidirectionally manipulate BNST^{CRF} neurons using a multiplexed Gi+Gq DREADD approach. c-d) Activation of the Gi-coupled KOR DREADD via systemic injection of Salvinorin B (SalB; 10 mg/kg) suppressed binge EtOH consumption compared to vehicle (VEH) injected controls (c; N's = 7 CON, 5 DREADD) but did not affect the % time in open arms of the elevated plus maze (EPM; left) or distance traveled (right; d; N's = 6 CON, 5 DREADD). e) DREADD virus hit map in the BNST (for Fig. 2a–f). Each dot is an individual hit and the grey Xs are a miss. Hit map was designed in Adobe Illustrator with an image adapted from The Mouse Brain Atlas (Franklin & Paxinos, 2007). f-g) Gq DREADD-mediated BNST^{CRF} neuron activation via clozapine-N-oxide (CNO; 5 mg/kg i.p.) did not change EtOH consumption (f; N's = 7 CON, 6 DREADD) or alter avoidance behavior on the open field (OF) measured via % time in center (left) but did reduce distance traveled (right; g; N's = 14 CON, 9 DREADD).*$P < 0.05$, unpaired t-tests between CON and DREADD; 2xANOVA main effects and interactions between groups and treatment; post hoc t-tests with H-S corrections as indicated. Data are presented as mean values +/- SEM. Detailed statistics are provided in Supplemental Table 1. Source data are provided as a Source Data file.

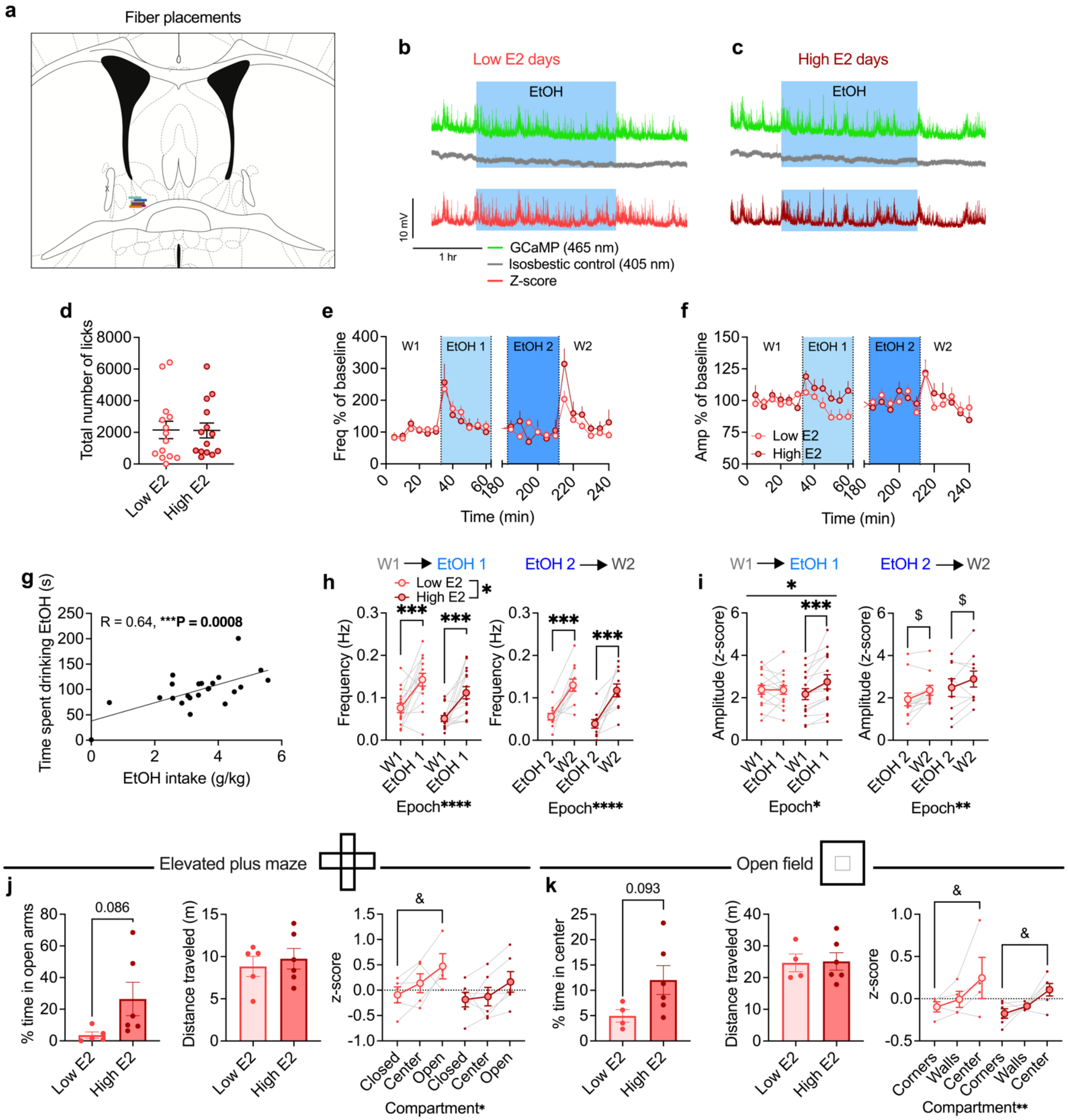

Supplementary Figure 3: Fiber photometry additional measures (related to Fig. 3). a) Hit map of fiber photometry cannulae in the BNST. Each line corresponds to the fiber placement and the grey X corresponds to a surgical miss that was not used in experiments due to poor GCaMP signal. Hit map was designed in Adobe Illustrator with an image adapted from The Mouse Brain Atlas (Franklin & Paxinos, 2007). b) Fiber photometry signals in the 465 nm excitation (GCaMP), isobestic 405 nm (control) wavelength channels, and normalized z-score during EtOH-DID in low E2 status and high E2 status female drinking days (c). d) There was no difference in total licks (TTLs) across epochs between low E2 and high E2 status drinking days (N = 5, 14 low E2 days, 14 high E2 days). e) Time course of transient event frequency as a % of W1 baseline across the 30 minutes of W1, first 30 minutes of EtOH (EtOH 1), last 30 minutes of EtOH (EtOH 2), and the first 30 minutes of W2 in 5 minutes bins. f) Time course of transient event peak amplitudes as a % of W1 baseline across the 30 minutes of W1, EtOH 1, EtOH 2, and the first 30 minutes of W2 in 5 minutes bins. g) There was a positive correlation between time spent drinking EtOH (s) and g/kg EtOH intake during the EtOH epoch (N = 5, 24 drinking days). h) There was an increase in transient event frequency during low and E2 status drinking days during the transition from the last 5 minutes of W1 to EtOH 1 (left; N = 5, 15 high E2 days, 15 low E2 days) and the last 5 minutes of EtOH 2 and W2 (right; N = 5, 11 low E2 days, 9

high E2 days; same Ns for i). i) There was an increase in transient event amplitude in the first 5 minutes of EtOH 1 compared to the last 5 minutes of W1during high E2 status drinking days but not during low E2 status drinking days (left), with no change during the transition from EtOH 2 to W2 (right). j) There was a trend towards increased % time spent in the open arms of the elevated plus maze (EPM) on high E2 status days compared to low E2 status days (left), with no change in distance traveled (center), and a trend towards increased normalized GCaMP signal in the open arms compared to the closed arms of the EPM on low E2 status days but not high E2 status days (right; N's = 5 low E2, 6 high E2). k) There was a trend towards increased % time spent in the center of the open field (OF) on high E2 status days (left), with no effect on distance (center), and a trend towards increased normalized GCaMP signal in the center compared to the corners of the OF on low and high E2 status days (right; N's = 4 low E2, 6 high E2). *$P < 0.05$, **$P < 0.01$, ***$P < 0.001$, ****$P < 0.0001$; unpaired t-tests between low vs high E2; 2xANOVA main effects and interactions between groups and time/compartment; post hoc t-tests with H-S corrections as indicated. $P < 0.10 for post hoc t-tests with H-S corrections between groups. Data are presented as mean values +/- SEM. Detailed statistics are provided in Supplemental Table 1. Source data are provided as a Source Data file.

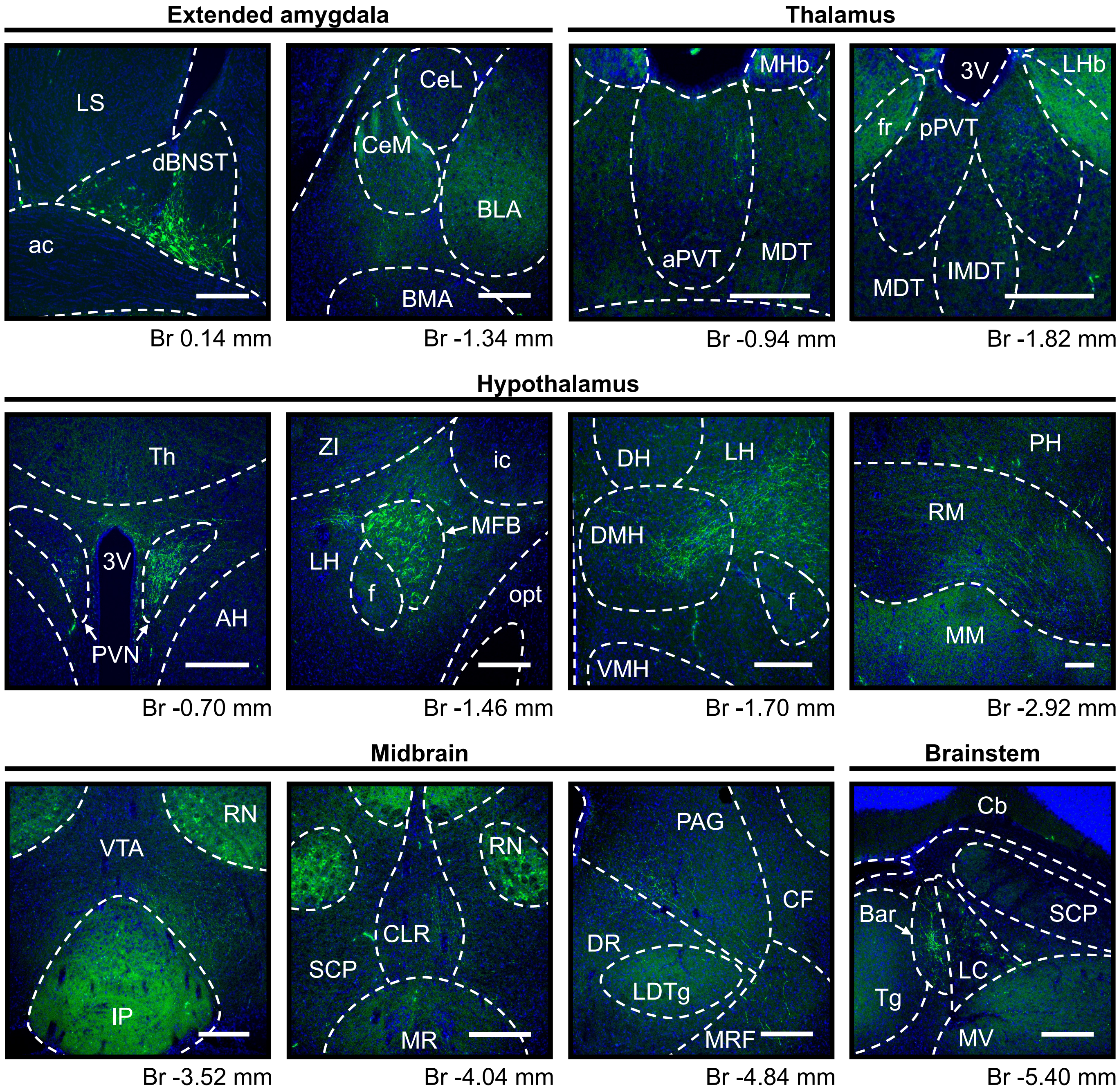

Supplementary Figure 4. BNST^{CRF} neuron projections (GCaMP tracing; related to Fig. 3). Female BNST^{CRF} neuron projection targets in the extended amygdala, thalamus, hypothalamus, midbrain, and brainstem. Abbreviations: 3V - 3rd ventricle, ac - anterior commissure, AH - anterior hypothalamic area, aPVT - paraventricular thalamic nucleus, anterior part, Bar - Barrington's nucleus, BLA - basolateral amygdala, BMA - basomedial amygdala, Cb - cerebellum, CeL - central amygdala, lateral division, CeM - central amygdala, medial division, CF - cuneiform nucleus, CLR - caudal linear nucleus of the raphe, dBNST - bed nucleus of stria terminalis, dorsal part, DMH - dorsomedial hypothalamic nucleus, DH - dorsal hypothalamic area, DR - dorsal raphe nucleus, f - fornix, fr - fasciculus retroflexus, ic - internal capsule, IMDT - intermediodorsal thalamic nucleus, IP - interpeduncular nucleus, LC - locus coeruleus, LDTg - laterodorsal tegmental nucleus, LH - lateral hypothalamus, LHb - lateral habenular nucleus, LS - lateral septal nucleus, MDT - mediodorsal thalamic nucleus, MDT - mediodorsal thalamic nucleus, MFB - medial forebrain bundle, MHb - medial habenular nucleus, MM - medial mammillary nucleus, MR - median raphe nucleus, MRF - mesencephalic reticular formation, MV - medial vestibular nucleus, opt - optic tract, PAG - periaqueductal gray, PH - posterior hypothalamic nucleus, pPVT - paraventricular thalamic nucleus, posterior part, PVN - paraventricular hypothalamic nucleus, RM - retromammillary nucleus, RN - red nucleus, SCP - superior cerebellar peduncle, Tg - tegmental nucleus, Th - thalamus, VMH - ventromedial hypothalamic nucleus, VTA - ventral tegmental area, ZI - zona incerta.

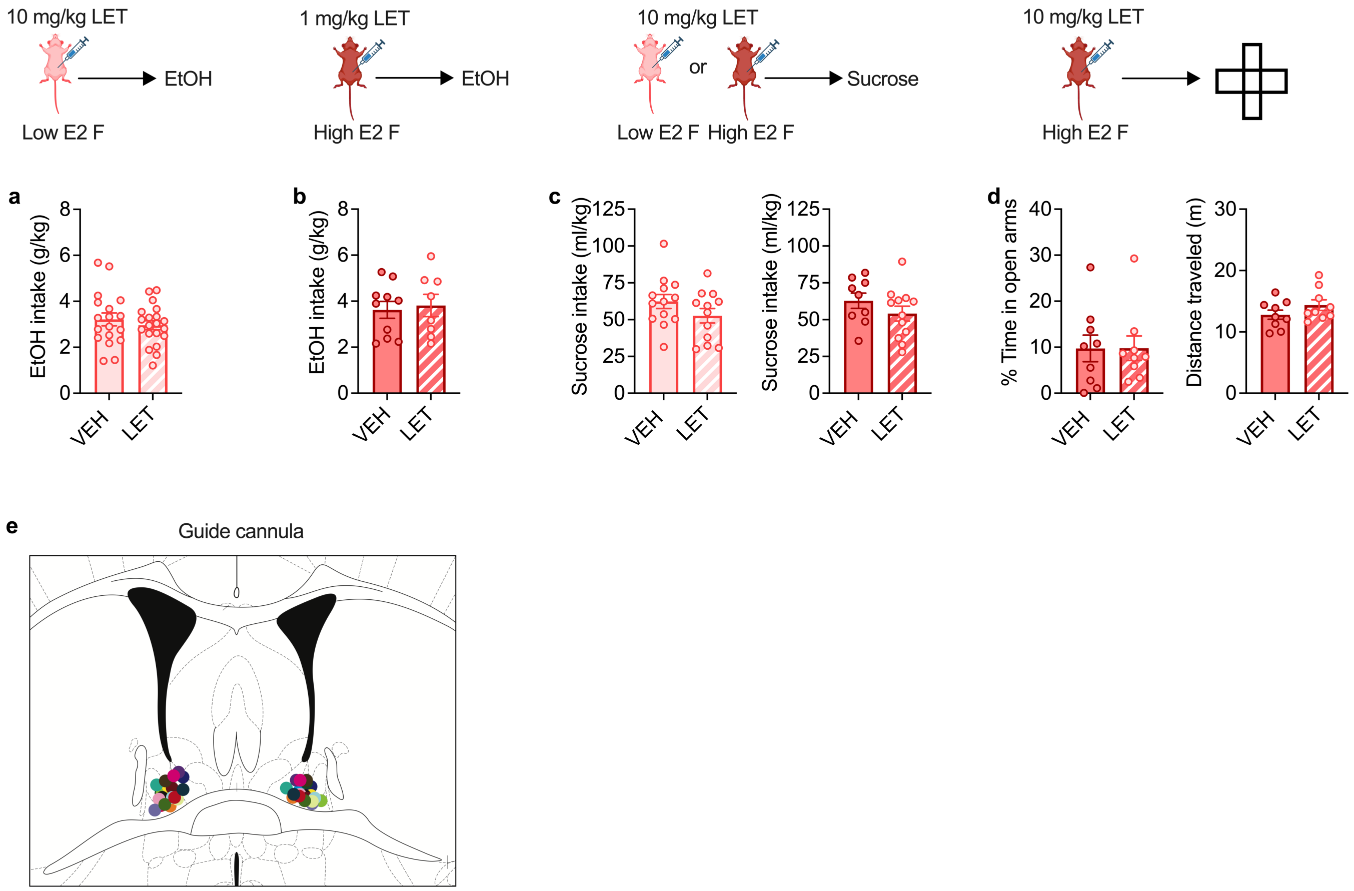

Supplementary Figure 5: Effects of rapid E2 signaling on binge alcohol consumption, binge sucrose consumption, and avoidance behavior are dose- and ovarian E2 state-dependent (related to Fig. 4). a-d) Behavioral effects of systemic estrogen synthesis inhibition using acute systemic administration of letrozole (LET) given 40 minutes prior to behavioral testing in high or low E2 status females. a) Acute LET (10 mg/kg i.p.) did not alter binge EtOH consumption in low E2 status females (N's = 18 VEH, 20 LET; Biorender license: XR27GW0F4J). b) Acute systemic administration of a low dose of LET (1 mg/kg, i.p.) did not alter binge EtOH consumption in high E2 status females, while a high dose of LET did (Fig. 4; N's = 10 VEH, 8 LET; Biorender license: UM27GVZ81B). c) Acute systemic LET (10 mg/kg, i.p) did not alter binge sucrose consumption in females in either a low ovarian E2 state (left, N's = 13 VEH, 12 LET) or high ovarian E2 state (right, N's = 9 VEH, 12 LET). d) Acute LET administration in high E2 status females 2 hours prior to EPM did not alter the % time spent in the open arms (left) or distance traveled (right; N's = 9 VEH, 9 LET). e) BNST guide cannulae placements following histology (for Fig. 4f-i); each dot is an individual hit. Hit map was designed in Adobe Illustrator with an image adapted from The Mouse Brain Atlas (Franklin & Paxinos, 2007). Data are presented as mean values +/- SEM/ Detailed statistics are provided in Supplemental Table 1. Source data are provided as a Source Data file.

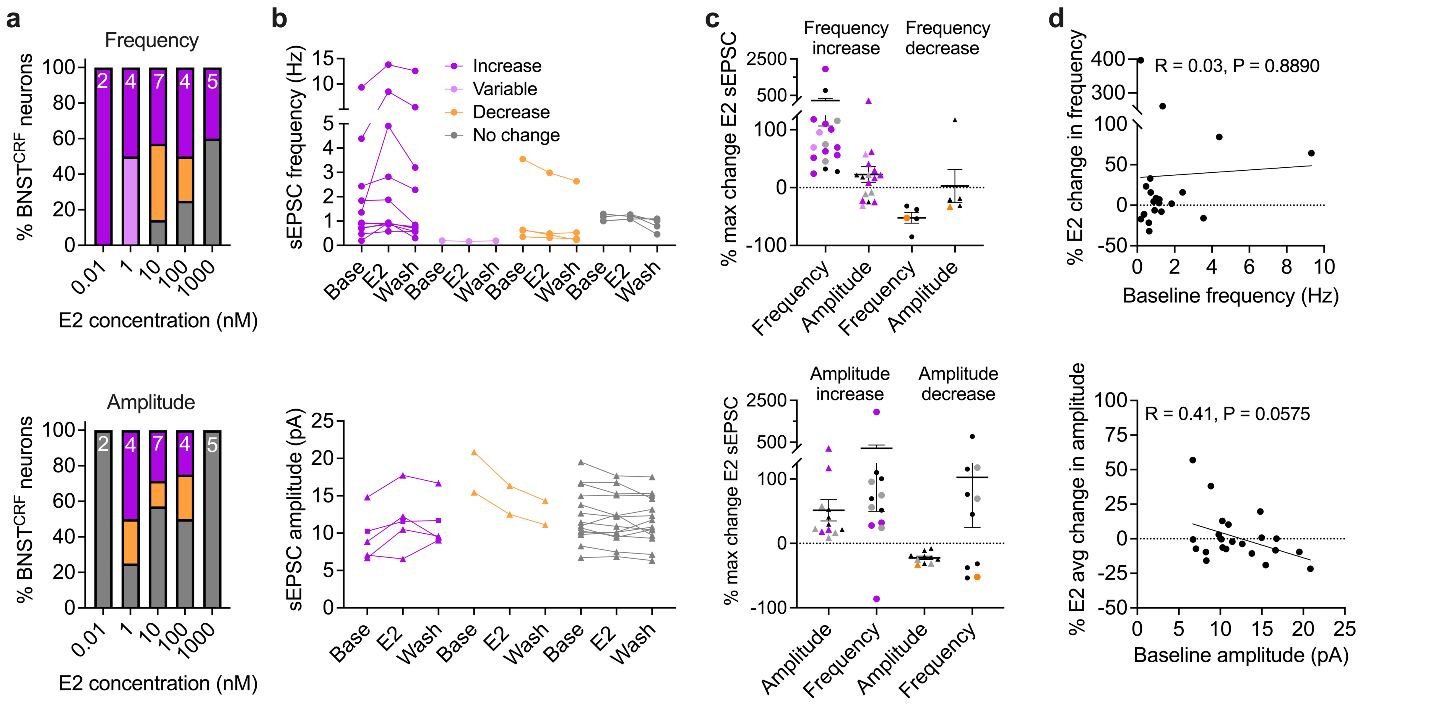

Supplementary Figure 6: E2 sEPSC frequency responsive BNST^{CRF} cells have a specific cellular phenotype (related to Fig. 3). a) Proportion of responder categories during 10-minute E2 wash on across E2 doses (0.01, 1, 10, 100, 1000 nM) for frequency (top) and amplitude (bottom). b) Raw average frequency (top) and amplitude (bottom) during baseline, E2 wash on, and washout for each cell. c) Maximum % change from baseline for cells categorized as increased and decreased during E2 wash on for frequency (top) and amplitude (bottom). Amplitude did not differ between BNST^{CRF} neurons that had increased vs. decreased frequency (top) and vice versa (bottom, N = 17 mice, 22 cells). d) % change from baseline in frequency (top) and amplitude (bottom) during E2 did not correlate with baseline frequency and amplitude. Data are presented as mean values +/- SEM. Detailed statistics are provided in Supplemental Table 1. Source data are provided as a Source Data file.

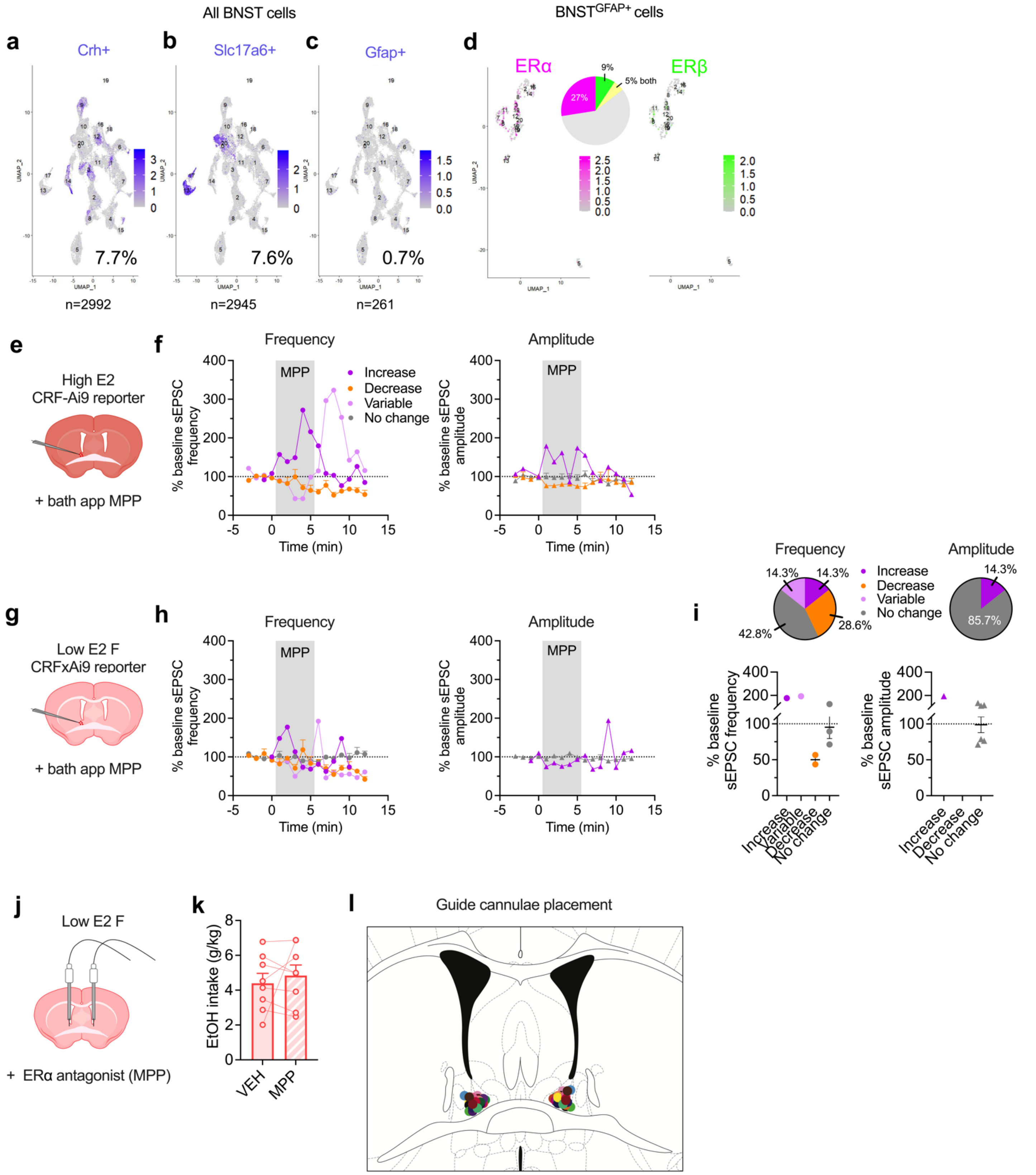

Supplementary Figure 7: BNST cell population analysis, MPP pharmacology (*ex vivo* slice electrophysiology and *in vivo* intra-BNST infusion; related to Fig. 5). a-d) Analysis of single nucleus RNA sequencing of female BNST nuclei (total cells: 38,806; GEO: GSE126836)[78]. a) *Crh* (CRF) expression (7.7%) in female BNST cells. b) *Slc117a6* (VGLUT2) expression (7.6%) in female BNST cells. c) *Gfap* (GFAP) expression (0.7%) in female BNST cells. d) ERα (27%) and ERβ (9%) expression (5% both) in female BNST^{GFAP} cells. e-f) Effects of acute bath application of the ERα antagonist methyl-piperidino-pyrazole (MPP) on excitatory synaptic transmission in BNST^{CRF} neurons during whole-cell slice electrophysiology recordings in high ovarian E2 female CRF-CrexAi9 reporter mice, as depicted in e (Biorender license: NF27GVWGQV). f) Time course of high E2 status BNST^{CRF} neurons that displayed an increase, decrease, or variable change in spontaneous

excitatory postsynaptic current (sEPSC) frequency (left) and amplitude (right) % change from baseline during the 5-minute MPP wash on application period and 5 minute washout. g-i) Effects of acute bath application of MPP on excitatory synaptic transmission in $BNST^{CRF}$ neurons during whole-cell slice electrophysiology recordings in low ovarian E2 female CRF-CrexAi9 reporter mice, as depicted in g (Biorender license: JF27GVZUJE). f) Time course of low E2 status $BNST^{CRF}$ neurons that displayed an increase, decrease, or variable change in spontaneous excitatory postsynaptic current (sEPSC) frequency (left) and amplitude (right) % change from baseline during the 5-minute MPP wash on application period and 5 minute washout. i) Bath application of MPP (3 μM) had no effect on spontaneous excitatory postsynaptic current (sEPSC) frequency (left) and amplitude (right) in a majority of cells during the 5 min wash on period (Ns = 3 mice, 7 cells). j) Depiction of strategy to site-deliver MPP (10 μM/200 nl/side or saline vehicle (VEH) to the BNST in low E2 females via bilateral indwelling cannulae 10 minutes prior to behavioral testing (Biorender license: BF27GVZGF6). k) ERα antagonism via intra-BNST MPP did not alter binge EtOH drinking in low ovarian E2 status females (Ns = 8 VEH, 8 MPP). l) BNST cannula hit map (for Fig. 4d-e, 5j-l); each dot is an individual hit. Hit map was designed in Adobe Illustrator with an image adapted from The Mouse Brain Atlas (Franklin & Paxinos, 2007). Data are presented as mean values +/- SEM. Detailed statistics are provided in Supplemental Table 1. Source data are provided as a Source Data file.

Table S1. Statistical details of all figures

Figure	number of samples and exclusions	Statistical methods and values
1B	Met: N = 9 (2 outliers excl); Pro: N = 9 (2 excl, irregular GAPDH band)	Unpaired two-tailed t-test between metestrus and proestrus (t12 = 2.39, *P = 0.0343).
1C	Met: N = 20; Pro: N = 20; each sample is pooled from 5 mice	Unpaired two-tailed t-test between metestrus and proestrus (t6 = 7.81, ***P = 0.0002).
1D	Met: N = 10; Pro: N = 9	All data log transformed (Y = ln[Y]); unpaired two-tailed t-test between metestrus and proestrus status females (t17 = 2.24, **P = 0.0390).
1F	F: N = 27 (representing Pro and non-Pro matched data points within cycle for each mouse when Pro was captured); M: N = 7; across 6 wks of alcohol DID	Mixed effects analysis: Week (F5,176 = 9.97, ****P < 0.0001), Group (F2,58 = 11.09, ****P < 0.0001), interaction (F10,176 = 0.73, P = 0.6996). Post hoc paired t-tests with Holm-Sidak corrections between low E2 and high E2 status females for each week (1: t42 = 2.45, P = 0.0552; 2: t42 = 3.81, **P = 0.0027; 3: t42 = 3.10,*P = 0.0172; 4: t42 = 2.27, P = 0.0561; 5: t42 = 2.92, P = *0.0225; 6: t42 = 1.24, P = 0.2226). Post hoc unpaired t-tests with Holm-Sidak corrections between low E2 status females and males for each week (1: t135 = 0.02, P = 0.9903; 2: t135 = 0.12, P = 0.9903; 3: t135 = 0.63, P = 0.8948; 4: t135 = 2.27, P = 0.1409; 5: t135 = 1.43, P = 0.5664; 6: t135 = 0.99, P = 0.7882).
1G	M: N = 11 ; low E2 F: N = 10 (1 excl, bottle leak), high E2 F: N = 9 (1 excl, bottle leak)	One-way ANOVA: Group (F2,25 = 5.04,*P = 0.0145). Post hoc unpaired t-tests with Holm-Sidak corrections between: low E2 status females and males (t25= 0.35, P = 0.7311); high E2 status females and males (t25= 2.69, *P = 0.0250); low E2 status and high E2 status females (t25= 2.89, *P = 0.0232).
1H	F: N = 15 (representing Pro and non-Pro matched data points within cycle for each mouse when Pro was captured); M: N = 15; across 6 wks of sucrose DID	Mixed effects analysis: Week (F5,158 = 1.59, P = 0.1674), Group (F2,42 = 0.91, P = 0.4099), interaction (F10,158 = 0.96, P = 0.4822).
1I	Males: N = 8 (1 excl, erroneously high consumption based on distributions); low E2 status females: N = 7, high E2 status females: N = 7 (1 excl, erroneously high consumption based on distributions)	One-way ANOVA: Group (F2,17 = 0.02, P = 0.9820).
1J	Males: N = 10; low E2 status females: N = 13, high E2 status females: N = 6	One-way ANOVA: Group (F2,26 = 4.98, *P = 0.0147). Post hoc unpaired t-tests with Holm-Sidak corrections between: low E2 status females and males (t26= 0.63, P = 0.5339); high E2 status females and males (t26= 2.46, *P = 0.0417); low E2 status and high E2 status females (t26= 3.11, *P = 0.0135).
1K	Males: N = 10; low E2 status females: N = 13, high E2 status females: N = 6	One-way ANOVA: Group (F2,26 = 1.29, P = 0.2926).
1L	Males: N = 10; low E2 status females: N = 12, high E2 status females: N = 7	One-way ANOVA: Group (F2,26 = 4.71, *P = 0.0179). Post hoc unpaired t-tests with Holm-Sidak corrections between: low E2 status females and males (t26= 0.26, P = 0.7989); high E2 status females and males (t26= 2.57, *P = 0.0321); low E2 status and high E2 status females (t26= 2.90, *P = 0.0225).
1M	Males: N = 10; low E2 status females: N = 12 (1 excl, freezing in open arms), high E2 status females: N = 7	One-way ANOVA: Group (F2,25 = 3.88, *P = 0.0340). Post hoc unpaired t-tests with Holm-Sidak corrections between: low E2 status females and males (t25 = 0.62, P = 0.5423); high E2 status females and males (t25 = 2.12, &P = 0.0863); low E2 status and high E2 status females (t25 = 2.72, *P = 0.0348).
1N	Males: N = 10; low E2 status females: N = 12 (1 excl, freezing in open arms), high E2 status females: N = 7	One-way ANOVA: Group (F2,25 = 0.62, P = 0.5450).
s1A	Dark: N = 20 (3 exl, acyclic w/ cycle greater than/equal to 8 days); light: N = 21 (1 exl, acyclic w/ cycle greater than/equal to 8 days)	Unpaired two tailed t-test between dark and light cycle females (t35 = 1.05, P = 0.3012).
s1D	Females: N = 27 (DID cycles 1-2: 8 excluded for zero days in proestrus or zero low E2 consumption; DID cycles 3-4: 6 excluded for zero low E2 consumption; DID cycles 5-6: 1 excluded for zero low E2 consumption	Simple linear regression: DID cycles 1-2: R = 0.248, P = 0.3062; DID cycles 3-4: R = 0.245, P = 0.2838; DID cycles 5-6: R = 0.247, P = 0.2241.
s1E	Males: N = 10; low E2 F: N = 13, high E2 F: N = 6	One-way ANOVA: Group (F2,26 = 8.07, **P = 0.0019). Post hoc unpaired t-tests with Holm-Sidak corrections between: low E2 status females and males (t26= 0.73, P = 0.4693); high E2 status females and males (t26= 3.17, **P = 0.0078); low E2 status and high E2 status females (t26= 3.94, P = 0.0016).
s1F	Males: N = 10; low E2 F: N = 12 (1 excl, freezing in open arms), high E2 F: N = 7	One-way ANOVA: Group (F2,25 = 4.71, *P = 0.0184). Post hoc unpaired t-tests with Holm-Sidak corrections between: low E2 status females and males (t25 = 1.30, P = 0.2050); high E2 status females and males (t25 = 1.86, P = 0.1447); low E2 status and high E2 status females (t25 = 3.07, *P = 0.0153).
2C	Low E2: N = 6, n = 16 cells (3 excl, noise); high E2: N = 7, n = 17 cells (4 excl, noise)	All data log transformed (Y = ln[Y]); unpaired two-tailed t-test between low E2 and high E2 status females (t24 = 3.44, **P = 0.0021).
2D	Low E2: N = 6, n = 16 cells (3 excl, noise); high E2: N = 7, n = 17 cells (4 excl, noise)	All data log transformed (Y = ln[Y]); unpaired two-tailed t-test between low E2 and high E2 status females (t24 = 0.29, P = 0.7748).
2E	Low E2: N = 6, n = 16 cells (3 excl, noise); high E2: N = 7, n = 17 cells (4 excl, noise)	All data log transformed (Y = ln[Y]); unpaired two-tailed t-test between low E2 and high E2 status females (t24 = 2.83, **P = 0.0092).
2G	Low E2: N = 6, n = 14 cells (2 excl, noise); high E2: N = 7, n = 14 cells (3 excl, noise)	All data log transformed (Y = ln[Y]); unpaired two-tailed t-test between low E2 and high E2 status females (t21 = 2.20, *P = 0.0395).
2H	Low E2: N = 6, n = 14 cells (2 excl, noise); high E2: N = 7, n = 14 cells (3 excl, noise)	All data log transformed (Y = ln[Y]); unpaired two-tailed t-test between low E2 and high E2 status females (t21 = 1.52, P = 0.1427).
2I	Low E2: N = 6, n = 14 cells (2 excl, noise); high E2: N = 7, n = 14 cells (3 excl, noise)	All data log transformed (Y = ln[Y]); unpaired two-tailed t-test between low E2 and high E2 status females (t21 = 1.12, P = 0.2740).
s2C	CON: N = 7, KORD: N = 7 (2 excl, misses)	2xRM-ANOVA: DREADD (F1,10 = 0.09, P = 0.7667), SalB (F1,10 = 5.24, *P = 0.0450), interaction (F1,10 = 4.41, P = 0.0620). Post hoc paired t-tests with Holm-Sidak corrections between VEH and SalB within group (CON: t10 = 0.15, P = 0.8864; KORD: t10 = 2.87, *P = 0.0328).
s2D	CON: N = 7 (1 excl, freezing in open arms), KORD: N = 7 (2 excl, misses)	Percent time in open arms: unpaired two-tailed t-test between CON and KORD (t13 = 0.06, P = 0.9502); distance traveled: unpaired two-tailed t-test between CON and KORD (t13 = 1.33, P = 0.2074).
s2F	CON: N = 8 (1 excl, VEH drinking < 1 g/kg), DREADD: N = 9 (3 excl: 2 misses, 1 VEH drinking < 1 g/kg)	2xRM-ANOVA: DREADD (F1,10 = 0.16, P = 0.7018), CNO (F1,10 = 0.81, P = 0.3895), interaction (F1,10 = 0.19, P = 0.6707).
s2G	CON: N = 14, DREADD: N = 14 (5 excl, misses)	Percent time in center: unpaired two-tailed t-test between CON and DREADD (t21 = 0.14, P = 0.8893); distance traveled: unpaired two-tailed t-test between CON and DREADD (t21 = 2.67, *P = 0.0142).
3D	N = 5, low E2 days = 15 (2 excl, technical error, high E2 status days = 15 (3 excl, technical error)	Unpaired two-tailed t-test between low E2 status and high E2 status females (t23 = 0.389, P = 0.7076).
3E	N = 5, low E2 days = 15 (1 excl, technical error), high E2 status days = 15 (1 excl, technical error)	Unpaired two-tailed t-test between low E2 status and high E2 status females (t26 = 3.24, **P = 0.0033).
3F	N = 5, low E2 days = 15 (3 excl: 1 technical error, 2 w/ 0 bouts), high E2 days = 15 (1 excl, technical error)	Unpaired two-tailed t-test between low E2 status and high E2 status females (t24 = 2.61, *P = 0.0153).
3H	N = 5, low E2 days = 15 (2 excl, trace technical error), high E2 days = 15	2xRM-ANOVA: Time (F2,52 = 13.58, ****P < 0.0001), Estrous (F1,26 = 0.28, P = 0.5993), interaction (F2,52 = 1.12, P = 0.3339). Post hoc paired t-tests with Holm-Sidak corrections between 5 s bins (1: 5 s prior to bout onset, 2: 0-5 s after bout onset, 3: 6-10 s after bout onset; 15 s total) within group: low E2 status (bin 1 vs bin 2: t52 = 1.94, P = 0.1115; bin 1 vs bin 3: t52 = 4.42, ***P = 0.0003; bin 2 vs bin 3: t52 = 2.48, P = 0.0639); high E2 status (bin 1 vs bin 2: t52 = 0.32, P = 0.7493; bin 1 vs bin 3: t52 = 2.74, *P = 0.0416; bin 2 vs bin 3: t52 = 2.42, P = 0.0639).
3I	N = 5, low E2 days = 15 (2 exc, trace technical error), high E2 days = 15	2xRM-ANOVA: Time (F2,38 = 1.63, P = 0.2097), Estrous (F1,19 = 0.29, P = 0.5945), interaction (F2,38 = 0.58, P = 0.5625).

3J	N = 5, drinking days: N = 30 (5 excl: 2 technical errors, 3 with <2 bouts)	Simple linear regression: R = 0.445, *P = 0.0257.
3K	Bouts: total mice: N = 5, low E2 status days: N = 15 (2 excl, 1 w/ bouts > 30 in a 30 min period, 1 technical error), high E2 status days: N = 15 (2 excl, 1 w/ bouts > 30 in a 30 min period, 1 technical error); Normalized GCaMP signal: N = 5, low E2 days = 15 (1 excl, technical error), high E2 days = 15 (1 excl, technical error)	Bouts (left): 2xRM-ANOVA: Epoch (F1,24 = 28.61, ****P < 0.0001), Estrous (F1,24 = 0.32, P = 0.5763), interaction (F1,24 = 6.86, *P = 0.0150). Post hoc paired t-tests with Holm-Sidak corrections between H2O and EtOH within group (low E2: t24 = 1.93, &P = 0.0655; high E2: t24 = 5.63, ****P < 0.0001). Normalized GCaMP signal (right): 2xRM-ANOVA: Epoch (F1,26 = 10.68, **P = 0.0030), Estrous (F1,26 = 0.15, P = 0.6979), interaction (F1,26 = 0.98, P = 0.3323). Post hoc paired t-tests with Holm-Sidak corrections between H2O and EtOH within group (low E2: t28 = 1.61, P = 0.1190; high E2: t28 = 3.19, *P = 0.0115).
3L	Bouts: N = 5, low E2 days = 15 (4 excl, technical error), high E2 days = 15 (6 excl: 5 technical error, 1outlier w/ > 30 bouts); Normalized GCaMP signal: N = 5, low E2 days = 15 (4 excl, technical error), high E2 days = 15 (6 excl, technical error)	Bouts: 2xRM-ANOVA: Epoch (F1,18 = 3.86, P = 0.0651), Estrous (F1,18 = 1.29, P = 0.2706), interaction (F1,18 = 0.77, P = 0.3912). Normalized GCaMP: 2xRM-ANOVA: Epoch (F1,18 = 1.59, P = 0.2226), Estrous (F1,18 = 1.24, P = 0.2791), interaction (F1,18 = 0.004, P = 0.9463).
3Q	N = 5, W1: low E2 days = 15, high E2 days = 15; EtOH 1: low E2 days = 15, high E2 days = 15; EtOH 2: low E2 days = 15 (4 excl, technical error), high E2 days = 15 (5 excl, technical error); W2: low E2 days = 15 (4 excl, technical error), high E2 days = 15 (4 excl, technical error)	RM Mixed-effects model: estrous (F1,28 = 0.40, P = 0.5286), epoch (F3,66 = 9.60, ****P < 0.0001), interaction (F3,66 = 0.86, P = 0.4652). Post hoc paired t-tests with Holm-Sidak corrections between epochs within group: low E2 status (W1 vs EtOH 1: t66 = 3.16, *P = 0.0142; W2 vs EtOH 2: t66 = 0.87, P = 0.6396); high E2 status (W1 vs EtOH 1: t66 = 2.95, *P = 0.0176; W2 vs EtOH 2: t66 = 3.03, *P = 0.0170).
3R	N = 5, W1: low E2 days = 15, high E2 days = 15; EtOH 1: low E2 days = 15, high E2 days = 15; EtOH 2: low E2 days = 15 (4 excl, technical error), high E2 days = 15 (5 excl, technical error); W2: low E2 days = 15 (4 excl, technical error), high E2 days = 15 (5 excl, technical error)	RM Mixed-effects model: estrous (F1,28 = 0.34, P = 0.5652), epoch (F3,66 = 1.40, P = 0.2499), interaction (F3,66 = 4.15, **P = 0.0094). Post hoc paired t-tests with Holm-Sidak corrections between epochs within group: low E2 status (W1 vs EtOH 1: t66 = 0.90, P = 0.9378; W2 vs EtOH 2: t66 = 0.00, P > 0.9999); high E2 status (W1 vs EtOH 1: t66 = 2.58, *P = 0.0480; W2 vs EtOH 2: t66 = 0.00, P > 0.9999).
s3D	N = 5, low E2 days = 15 (1 exc, technical error), high E2 days = 15 (1 exc, technical error)	Unpaired two-tailed t-test between low E2 status and high E2 status females (t26 = 0.04, P = 0.9664).
s3G	N = 5, drinking days = 30 (6 excl, technical error, 1 excl due to lack of dummy drip EtOH data)	Simple linear regression: R = 0.64, ***P = 0.0008.
s3H	N = 5, W1 to EtOH 1 (right): low E2 = 15, high E2 = 15; EtOH 2 to W2 (left): low E2 = 15 (4 excl, tehcnical error), high E2 = 15 (6 excl, technical error)	W1 to EtOH 1: 2xRM-ANOVA: Epoch (F1,28 = 32.84, ****P = < 0.0001), Estrous (F1,28 = 4.58, *P = 0.0412), interaction (F1,28 = 0.07, P = 0.7978). Post hoc paired t-tests with Holm-Sidak corrections between epochs (low E2: t28 = 4.24, ***P = 0.0004; high E2: t28 = 3.89, ***P = 0.0006). EtOH 2 to W2: 2xRM-ANOVA: Epoch (F1,19 = 48.90, ****P < 0.0001), Estrous (F1,19 = 1.22, P = 0.2841), interaction (F1,19 = 0.04, P = 0.8414). Post hoc paired t-tests with Holm-Sidak corrections between epochs (low E2: t19 = 4.92, $P = 0.0002; high E2: t19 = 4.97, $P = 0.0002).
s3I	N = 5, W1 to EtOH 1 (right): low E2 = 15, high E2 = 15; EtOH 2 to W2 (left): low E2 = 15 (4 excl, tehcnical error), high E2 = 15 (6 excl, technical error)	W1 to EtOH 1: 2xRM-ANOVA: Epoch (F1,28 = 6.83, *P = 0.0143), Estrous (F1,28 = 0.06, P = 0.8036), interaction (F1,28 = 7.53, *P = 0.0105). Post hoc paired t-tests with Holm-Sidak corrections between epochs (low E2: t28 = 0.09, P = 0.9266; high E2: t28 = 3.79, **P = 0.0015). EtOH 2 to W2: 2xRM-ANOVA: Epoch (F1,18 = 8.49, **P = 0.0093), Estrous (F1,18 = 1.25, P = 0.2777), interaction (F1,18 = 0.09, P = 0.7678). Post hoc paired t-tests with Holm-Sidak corrections between epochs (low E2: t18 = 0.09, $P = 0.0546; high E2: t18 = 3.79, $P = 0.0950).
s3J	Low E2: N = 6 (1 mouse did not enter open arms), high E2: N = 6	Left: percent time in open arms: unpaired two-tailed t-test between low E2 status and high E2 status females (t9 = 1.93, &P = 0.0857); distance traveled: unpaired two-tailed t-test between low E2 status and high E2 status females (t9 = 0.52, P = 0.6143). Right: RM Mixed-effects model: compartment (F2,17 = 6.48, **P = 0.0081), estrous (F1,9 = 1.14, P = 0.3133), interaction (F2,17 = 0.44, P = 0.6526). Post hoc paired t-tests with Holm-Sidak corrections between compartments within group: low E2 status (closed arms vs open arms: t17 = 2.09, P = &0.0579; closed arms vs center: t17 = 1.23, P = 0.4147; open arms vs center: t17 = 1.76, P = 0.3314); high E2 status (closed arms vs open arms: t17 = 2.05, P = 0.2490; closed arms vs center: t17 = 0.35, P = 0.7323; open arms vs center: t17 = 1.71, P = 0.3314)
s3K	Low E2: N = 4, high E2: N = 6	Left: percent time in center: unpaired two-tailed t-test between low E2 status and high E2 status females (t8 = 1.91, &P = 0.0927); distance traveled: unpaired two-tailed t-test between low E2 status and high E2 status females (t8 = 0.11, P = 0.9160). Right: 2xRM-ANOVA: compartment (F2,16 = 6.98, **P = 0.0066), estrous (F1,8 = 1.02, P = 0.3432), interaction (F2,16 = 0.08, P = 0.9219). Post hoc paired t-tests with Holm-Sidak corrections between compartments within group: low E2 status (walls vs corners: t16 = 0.66, P = 0.5158; walls vs center: t16 = 1.90, P = 0.1447; corners vs center: t16 = 2.57, &P = 0.0607); high E2 status (walls vs corners: t16 = 0.80, P = 0.4356; walls vs center: t16 = 1.78, P = 0.1779; corners vs center: t16 = 2.58, &P = 0.0587).
4B	VEH: N = 17 (4 excl, VEH drinking < 1 g/kg) , LET: N = 20 (1 excl, matched VEH exclusion mice)	Unpaired two-tailed t-test between VEH and LET treatment (t30 = 3.68, ***P = 0.0009).
4C	VEH: N = 16 (1 excl, fell off maze), LET: N = 15 (1 excl, fell off maze)	Percent time in open arms: unpaired two-tailed t-test between VEH and LET treatment (t27 = 0.09, P = 0.9309); distance traveled: unpaired two-tailed t-test between VEH and LET treatment (t27 = 0.16, P = 0.8704).
4E	VEH: N = 7, LET: N = 6	1 hr DID: unpaired two-tailed t-test between VEH and E2 treatment (t11 = 0.67, P = 0.5183); 2 hr DID: unpaired two-tailed t-test between VEH and E2 treatment (t11 = 0.74, P = 0.4724).
4G	VEH: N = 8, E2: N = 8 (1 excl, surgial miss)	1 hr DID: unpaired two-tailed t-test between VEH and E2 treatment (t13 = 3.45, P = **0.0043); 2 hr DID: unpaired two-tailed t-test between VEH and E2 treatment (t13 = 2.90, *P = 0.0125).
4H	VEH: N = 13 (1 excl, VEH drinking < 1 g/kg), mem-E2: N = 13 (2 excl, surgical misses)	1 hr DID: unpaired two-tailed t-test between VEH and E2 treatment (t21 = 2.46, P = *0.0227); 2 hr DID: unpaired two-tailed t-test between VEH and E2 treatment (t21 = 1.74, &P = 0.0968).
4I	VEH: N = 8 (1 excl, surgical miss), E2: N = 9 (1 excl, surgical miss)	Percent time in open arms: unpaired two-tailed t-test between VEH and E2 treatment (t13 = 0.55, P = 0.5909); distance traveled: unpaired two-tailed t-test between VEH and E2 treatment (t13 = 0.13, P = 0.8986).
4K	0.01 nM E2: N = 2, n = 2 cells; 1 nM E2: N = 4, n = 4 cells; 10 nM E2: N = 5, n = 7 cells, 100 nM E2: N = 4, n = 4 cells, 1000 nM E2 application: N = 3, n = 5 cells, 100 nM mE2: N = 3, n = 6 cells	2xRM-ANOVA: category (frequency vs amplitude; F1,44 = 2.65, P = 0.1105), E2 dose (F5,44 = 1.29, P = 0.2826), interaction (F5,44 = 0.87, P = 0.5070).
4L,M	N = 17 mice, n = 22 cells; Freq: n = 13 increase in freq, n = 11 no increase in freq; Amp: n = 5 increase in amp, n = 17 no increase in amp	Fisher's exact test (P = *0.0305).
s5A	VEH: N = 19 (1 excl, veh drinking < 1 g/kg), LET: N = 20	Unpaired two-tailed t-test between VEH and LET treatment (t36 = 0.75, P = 0.4569).
s5B	VEH: N = 10, LET: N = 8	Unpaired two-tailed t-test between VEH and LET treatment (t16 = 0.32, P = 0.7521).
s5C	Low E2: VEH: N = 13, LET: N = 12; high E2: VEH: N = 9, LET: N = 12	Low E2: unpaired two-tailed t-test between VEH and LET treatment (t23 = 1.42, P = 0.1686); high E2: unpaired two-tailed t-test between VEH and LET treatment (t19 = 1.18, P = 0.2494).
s5D	VEH: N = 9, LET: N = 9	Percent time in open arms: unpaired two-tailed t-test between VEH and LET treatment (t16 = 0.01, P = 0.9912); distance traveled: unpaired two-tailed t-test between VEH and LET treatment (t16 = 1.36, P = 0.1922).
s6D	N = 17 mice, n = 22 cells	Freqency: simple linear regression: R = 0.03, P = 0.8835; amplitude: simple linear regression: R = 0.41, P = 0.0575.
5D	N = 4	2xRM-ANOVA: receptor (F1,3 = 36.64, **P = 0.0090), CRF status (F1,3 = 9.74, P = 0.0524), interaction (F1, 3 = 3.90, P = 0.1428). Post hoc paired t-tests with Holm-Sidak corrections between ERα and ERβ: CRF+ (t3 = 11.98, **P = 0.0025); CRF- (t3 = 9.19, **P = 0.0054). Post hoc paired t-tests with Hold-Sidak corrections between CRF+ and CRF-: ERα (t6 = 3.64, *P = 0.0216); ERβ (t6 = 2.13, &P = 0.0775).
5E	N = 4	2xRM-ANOVA: receptor (F1,3 = 26.81, *P = 0.0140), vGLUT2 status (F1,3 = 15.71, *P = 0.0287), interaction (F1,3 = 18.72, *P = 0.0228). Post hoc paired t-tests with Holm-Sidak corrections between ERα and ERβ: vGLUT2+ (t3 = 8.18, **P = 0.0076); vGLUT2- (t3 = 2.06, P = 0.2463). Post hoc paired t-tests with Hold-Sidak corrections between vGLUT2+ and vGLUT-: ERα (t6 = 5.65, **P = 0.0026); ERβ (t6 = 1.08, P = 0.3211).
5H	N = 4 mice, n = 7 cells (frequency: 1 increase, 1 variable change, 5 decrease; ampltiude: 1 increase, 2 decrease, 4 no change)	One sample t-test: frequency (increase: too few points; variable: too few points; decrease: t4 = 7.66, **P = 0.0016); amplitude (increase: too few points; too few points; no change: t4 = 1.63, P = 0.1841).
5J	VEH: N = 11 (3 excl, 2 misses, 1 no dye/damage), MPP: N = 11 (3 excl, 2 misses, 1 no dye/damage); VEH: N = 9 (2 excl, 1 miss, 1 no dye/damage), PHTPP = 9 (2 excl, 1 miss, 1 no dye/damage)	MPP: paired two-tailed t-test between VEH and MPP treatment (t7 = 2.91, *P = 0.0227); PHTPP: paired two-tailed t-test between VEH and PHTPP treatment (t6 = 1.03, P = 0.3435)
5K	VEH: N = 8, MPP: N = 8 (1 excl, mouse died before histology)	Percent time in light: unpaired two-tailed t-test between VEH and MPP treatment (t13 = 0.77, P = 0.4526); distance in light: upaired t-test between VEH and MPP treatment (t13 = 1.41, P = 0.1810)
5L	VEH: N = 6 (1 excl, mouse died before histology), PHTPP: N = 6	Percent time in center: unpaired two-tailed t-test between VEH and PHTPP treatment (t9 = 0.08, P = 0.9369); distance in light: upaired t-test between VEH and PHTPP treatment (t9 = 0.98, P = 0.3545)
s7I	N = 3 mice, n = 7 cells (frequency: 1 increase, 1 variable change, 2 decrease, 3 no change; ampltiude: 1 increase, 6 no change)	One sample t-test: frequency (increase: too few points; variable: too few points; decrease: t1 = 7.66, P = 0.0826; no change: t2 = 0.29, P = 0.7964); amplitude (increase: too few points; no change: t5 = 0.10, P = 0.9249).
s7K	VEH: N = 9 (1 excl, did not receive paired MPP), MPP: N = 8	Paired two-tailed t-test between VEH and MPP treatment (t7 = 0.65, P = 0.5375).
